# Ethanol itself is a holoprosencephaly-inducing teratogen

**DOI:** 10.1371/journal.pone.0176440

**Published:** 2017-04-25

**Authors:** Mingi Hong, Robert S. Krauss

**Affiliations:** Department of Cell, Developmental, and Regenerative Biology, Icahn School of Medicine at Mount Sinai, New York, NY, United States of America; University of Kentucky, UNITED STATES

## Abstract

Ethanol is a teratogen, inducing a variety of structural defects in developing humans and animals that are exposed in utero. Mechanisms of ethanol teratogenicity in specific defects are not well understood. Oxidative metabolism of ethanol by alcohol dehydrogenase or cytochrome P450 2E1 has been implicated in some of ethanol’s teratogenic effects, either via production of acetaldehyde or competitive inhibition of retinoic acid synthesis. Generalized oxidative stress in response to ethanol may also play a role in its teratogenicity. Among the developmental defects that ethanol has been implicated in is holoprosencephaly, a failure to define the midline of the forebrain and midface that is associated with a deficiency in Sonic hedgehog pathway function. Etiologically, holoprosencephaly is thought to arise from a complex combination of genetic and environmental factors. We have developed a gene-environment interaction model of holoprosencephaly in mice, in which mutation of the Sonic hedgehog coreceptor, Cdon, synergizes with transient in utero exposure to ethanol. This system was used to address whether oxidative metabolism is required for ethanol’s teratogenic activity in holoprosencephaly. We report here that *t*-butyl alcohol, which is neither a substrate nor an inhibitor of alcohol dehydrogenases or Cyp2E1, is a potent inducer of holoprosencephaly in *Cdon* mutant mice. Additionally, antioxidant treatment did not prevent ethanol- or *t*-butyl alcohol-induced HPE in these mice. These findings are consistent with the conclusion that ethanol itself, rather than a consequence of its metabolism, is a holoprosencephaly-inducing teratogen.

## Introduction

Holoprosencephaly (HPE) is a common congenital disorder in which the midline of the forebrain and/or midface is lacking [1]. HPE occurs with a frequency of about 1 in 250 conceptions, with ~97% of holoprosencephalic fetuses succumbing in utero [2] [3]. Presentation of HPE is extremely variable, with a spectrum of phenotypes ranging from failure to partition the forebrain into hemispheres to deficits in the midfacial midline [4, 5]. The most severe cases are not compatible with survival. However, cases with mild forebrain involvement are associated with mental deficiency [6, 7].

Both genetic and environmental factors are implicated in the etiology of HPE [5, 6, 8–10]. Heterozygous, loss-of-function mutations in the Sonic hedgehog (Shh) signaling pathway are associated with HPE [10]. However, some mutation carriers have no obvious clinical manifestation, even in affected pedigrees [10–12]. In contrast, offspring who inherit such mutations are at high risk of HPE. Modeling of these observations has led to a multifactorial, “mutation plus modifier” paradigm, in which the phenotypic outcome associated with a heterozygous mutation is influenced by more common genetic variants and/or environmental exposures [13]. Among the non-genetic risk factors implicated in HPE is fetal alcohol exposure [8, 9]. Exposure to specific teratogens may be sufficient to cause HPE in some cases [14, 15]. However, it is likely that many structural birth defects are caused by a complex combination of genetic and environmental factors, which interact to disrupt morphogenetic events during development [16, 17].

We have modeled this type of phenomenon in mice. Cdon is coreceptor for Shh, binding directly to Shh and to other components of the Shh receptor, including the primary receptor, PTCH1, and the additional coreceptors, Boc and Gas1 [18–20]. Heterozygous, loss-of-function mutations in *CDON* have been identified in some HPE patients [18]. *Cdon* mutant mice develop HPE in a strain-dependent manner [21, 22]. *Cdon* mutants on a 129S6 background display only mild, mid-facial features of HPE with low penetrance. These mice have a sub-threshold defect of Shh signaling and are sensitized to HPE-modifying factors, including dosage-dependent loss of one of the other Shh coreceptor-encoding genes (*Gas1* or *Boc*) [23, 24]. Significantly, 129S6 *Cdon*^-/-^ mice developed a wide spectrum of HPE phenotypes, at high penetrance, upon in utero exposure to ethanol (EtOH); in contrast, wild type and heterozygous littermates did not display HPE [25].

Mechanisms of EtOH teratogenicity are not well understood. EtOH is oxidized to acetaldehyde by alcohol dehydrogenase (ADH), and acetaldehyde is oxidized to acetate by aldehyde dehydrogenase (ALDH). Acetate enters the carbon pool with some excreted as CO_2_ [26]. Many of EtOH’s toxic effects involve its metabolism by ADH and/or cytochrome P450 2E1 (Cyp2E1, which also produces acetaldehyde), with ADH responsible for the great majority of EtOH breakdown [26, 27]. Several potential mechanisms of ethanol teratogenesis require its oxidative metabolism. First, EtOH-derived acetaldehyde has been implicated in EtOH-induced exencephaly [28]. A second possible mechanism involves interference with retinoic acid (RA) synthesis. Similar to EtOH metabolism, vitamin A (retinol) is converted to RA by a two-step ADH/ALDH mechanism. EtOH and acetaldehyde may act as competitive inhibitors of the ADH/ALDH enzymes involved in RA production, with a resulting failure to produce sufficient levels of RA for normal developmental patterning [29–31]. Finally, while Cyp2E1 is quantitatively less important than ADH, EtOH metabolism by this enzyme produces both acetaldehyde and reactive oxygen species (ROS) [26, 27]. Generalized oxidative stress to exposed fetuses may therefore also contribute to EtOH-induced HPE. Mechanisms that do not require EtOH metabolism also exist. EtOH itself perturbs cellular membranes, and it can also bind and inhibit the function of specific membrane proteins, such as the cell adhesion molecule, L1 [32–36].

**Fig 1:**
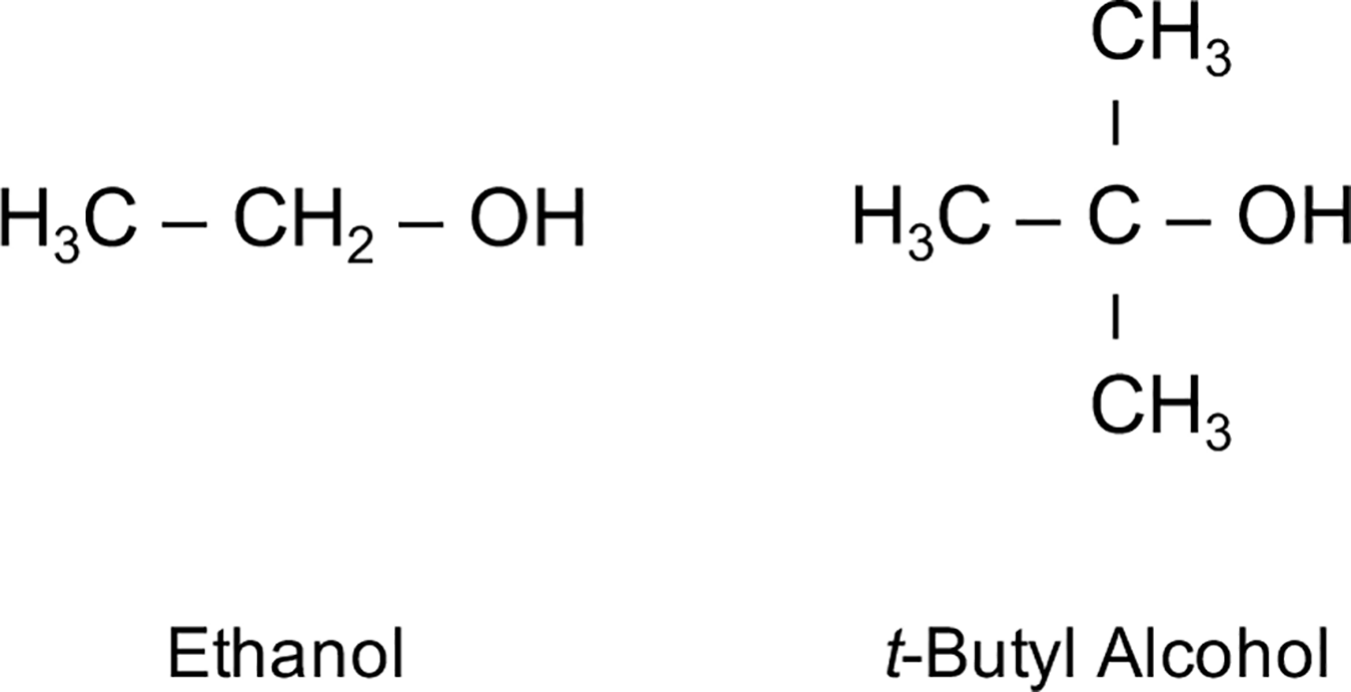
Structures of ethanol and *t*-butyl alcohol.

To begin to assess mechanisms of fetal alcohol-induced HPE, we tested the ability of *t*-butyl alcohol (Fig 1; IUPAC name, 2-Methylpropan-2-ol; abbreviated here as *t*-BuOH) to induce HPE in 129S6 *Cdon* mutant mice. *t*-BuOH is: 1) neither a substrate nor an inhibitor of ADHs or Cyp2E1; 2) poorly metabolized by oxidative processes; and 3) excreted mainly as the sulfate conjugate of the alcohol group [37–39]. Additionally, we tested the ability of antioxidants to influence EtOH-induced HPE in 129S6 *Cdon* mutant mice. We find that *t*-BuOH is a potent inducer of HPE, and that antioxidant treatment is not effective in preventing EtOH-induced HPE, in these mice. These findings are consistent with the notion ethanol itself, rather than a consequence of its metabolism, is an HPE-inducing teratogen.

## Materials and methods

### Mice

This study was carried out in strict accordance with the recommendations in the Guide for the Care and Use of Laboratory Animals of the National Institutes of Health. The protocol was approved by the Icahn School of Medicine at Mount Sinai Institutional Animal Care and Use Committee (IACUC). Our animal facility is accredited by the Association for Assessment and Accreditation of Laboratory Animal Care International (AAALAC).

Two- to three-month-old Cdon^+/tm1Rsk^ (Cdon^+/-^) mice on a 129S6/SvEvTac (129S6) background were mated for one hour in the dark and plugged females were collected. The time of the plug was designated as embryonic day (E) 0.0. EtOH administration was performed as described [25]. For *t*-BuOH treatment, pregnant female mice were injected intraperitoneally with 1g/kg of *t*-BuOH in saline at E7.0 and again 4 hours later. Saline injections were used as a control. We used the protocol described by Hirota et al. for antioxidant treatment [40]. Briefly, N-acetylcysteine (50 mg/kg body weight) and α-tocopherol (TCP, 1g/kg body weight) were given intraperitoneally at E5.0, E6.0 and E7.0. As an indicator of oxidative stress after alcohol treatment, total reactive oxygen species (ROS)/reactive nitrogen species (RNS) in the liver were analyzed with the OxiSelect *In vitro* ROS/RNS Assay Kit (Cell Biolabs) as per the manufacturer’s instructions. Briefly, the assay measures ROS/RNS-mediated formation of the fluorescent product 2’,7’-dichlorodihydro-fluorescein (DCF) from a starting fluorogenic probe, 2’,7’-dichlorodihydrofluorescein DiOxyQ (DCFH-DiOxyQ). DCF fluorescence (λ_ex_ = 480 nm, λ_em_ = 530 nm) is proportional to the amount of ROS/RNS in the sample. Measurements were performed on a SpectraMax i3x microplate reader (Molecular Devices). Livers were homogenized on ice and centrifuged at 10,000g for 5 min. Protein concentrations were analyzed by Bradford assay. Liver GSH levels were measured using the GSH-Glo^TM^ Glutathione Assay (Promega) following the manufacturers protocol. Livers were harvested 12 hours after the initial dose of alcohol.

### Histology and whole mount in situ hybridization

Embryos were dissected out and fixed overnight in 4% paraformaldehyde in PBS. They were then dehydrated through a graded ethanol series, embedded in paraffin and sectioned at 8 μm. H&E staining was performed as described [25]. Slides were then dehydrated through graded ethanol and xylene and mounted with Permount (Fisher Scientific).

For whole-mount RNA in situ hybridization, E10.0 embryos were prepared essentially as described previously [41], except that they were treated with 10 μg/ml proteinase K (QIAGEN) in phosphate-buffered saline, 0.1% Tween-20 (PBT) for 45 minutes. Embryos were rinsed, postfixed, and hybridized with digoxygenin-labeled probe in hybridization mix [50% formamide, 1.3x SSC, 5 mM EDTA, 50 μg /ml yeast RNA, 0.2% Tween 20, 0.5% 3-[(3-cholamidopropyl) dimethylammonio] propanesulfonate, and 100 μg /ml heparin] overnight at 65°C. After washing and blocking, embryos were incubated overnight with alkaline phosphatase-conjugated anti-digoxigenin antibody (1:2000; Roche) in blocking buffer (2% blocking reagent [Roche]), 20% heat-inactivated lamb serum in 100 mM maleic acid, pH 7.5, 150 mM NaCl, and 0.1% Tween 20 [MABT]). After washes in Tris-buffered saline with 0.1% Tween-20 (TBST) and 100 mm NaCl, 100 mm Tris-HCl, pH 9.5, 50 mm MgCl2, and 0.1% Tween -20 (NTMT), signals were developed using BM Purple AP Substrate (Roche).

## Results

### *t*-BuOH induces HPE in *Cdon* mutant mice

To test whether oxidative catabolism is a critical feature of EtOH-induced HPE, we asked whether an alcohol congener not subject to such metabolism induces HPE in 129S6 *Cdon* mutant mice (hereafter simply called *Cdon* mutant mice). *t*-BuOH is not effectively metabolized by ADH or Cyp2E1 [37–39], so it was used in place of EtOH in our standard protocol [25]. Briefly, one-hour timed matings were set between *Cdon* heterozygotes, and pregnant females were treated IP with either *t*-BuOH in saline, or saline alone as a vehicle control, at E7.0 and again four hours later. A dose of 2 g/kg (*t*-BuOH/body weight) resulted in maternal lethality. A dose of 1 g/kg produced a spectrum of HPE phenotypes in *Cdon*^*-/-*^ mice that was qualitatively and quantitatively similar to our standard protocol with EtOH (see below), and was not associated with any lethality, so this dose was used for detailed analysis. It should be noted that, on a moles-delivered basis, this dose of *t*-BuOH is ~18% that of EtOH in this model (1.0 vs. 3.48 g/kg, respectively, with the MW of *t*-BuOH = 74 and EtOH = 46).

Initial analyses were performed on embryos collected at E10.0. Similar to EtOH-treated embryos [25], *t*-BuOH-treated embryos had two or three fewer somite pairs at this stage than saline-treated controls, irrespective of genotype (Table 1). Therefore, as seen with in utero exposure to EtOH, *t*-BuOH caused a slight developmental delay that was independent of *Cdon* status. Approximately 15% of EtOH-treated *Cdon*^*-/-*^ embryos displayed severe forms of HPE visible at E10.0 [25]. These embryos were characterized as having a small forebrain that failed to partition, either fully or partially, into left and right hemispheres (these embryos died in utero and were resorbed before E12.0). A similar percentage (19%) of *t*-BuOH-treated *Cdon*^*-/-*^ embryos also showed a severe forebrain HPE phenotype (Table 1, and see below). In contrast, wild type and *Cdon*^*+/-*^ littermates were not affected by *t*-BuOH treatment, nor were saline-treated controls of any genotype.

**Table 1:** Effects of *t*-BuOH treatment on embryos at E10.0.

Defect	Treatment	Genotype (# affected/total (%))	
		*Cdon*^*+/-*^	*Cdon*^*-/-*^
External forebrain defects	Saline	0/12	0/10
	*t*-BuOH	0/73	12/63 (19%)*
# somites	Saline	33.5 ± 1.45	33.5 ± 1.84
	*t*-BuOH	30.48 ± 3.05**	31.0 ± 2.22**

*p<0.0001 when comparing *t*-BuOH-treated *Cdon*^*-/-*^ embryos with *t*-BuOH-treated *Cdon*^*+/-*^ embryos with two-tailed Fisher’s exact test.

**p<0.001 when comparing *t*-BuOH-treated embryos with saline-treated embryos of the same genotype using Student’s t-test. *t*-BuOH-treated embryos showed delayed somite formation irrespective of genotype.

**Fig 2:**
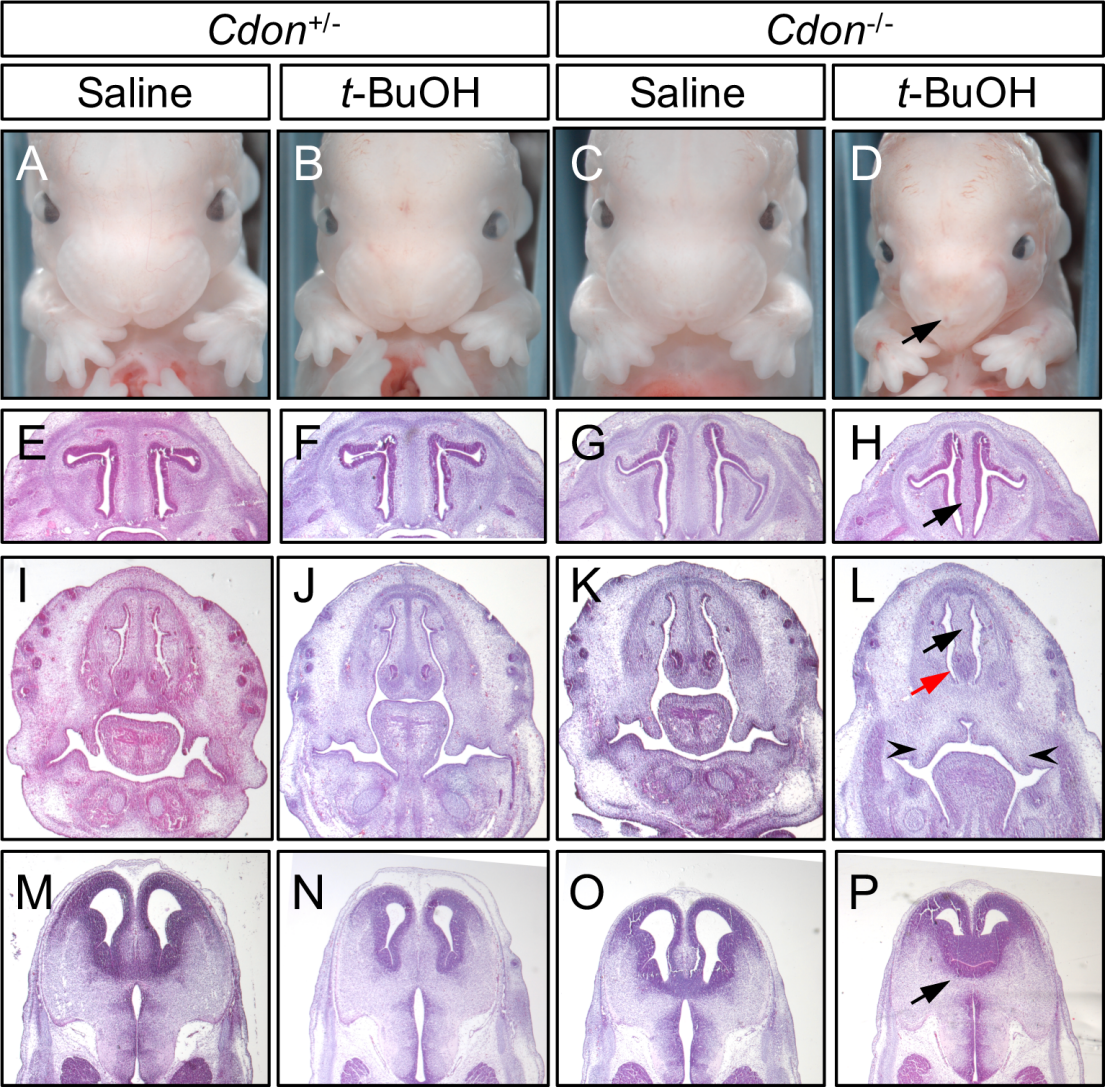
*t*-BuOH induces HPE in *Cdon* mutant mice. (A-D) Frontal views of E14 embryos. *t*-BuOH-treated *Cdon*^*-/-*^ embryos (D) developed strong facial features of HPE, including single nostril (arrow). (E-P) H&E stained coronal sections of E14 embryos. Midfacial and forebrain midline structures were missing or reduced in *t*-BuOH-treated *Cdon*^*-/-*^ embryos, including cartilage primordium of the nasal septum (H, arrow); nasal septum (L, black arrow); vomeronasal organ (L, red arrow); defective palatal shelves (L, arrowheads) flanking midline cleft; and ventral diencephalon midline structure (P, arrow).

**Fig 3:**
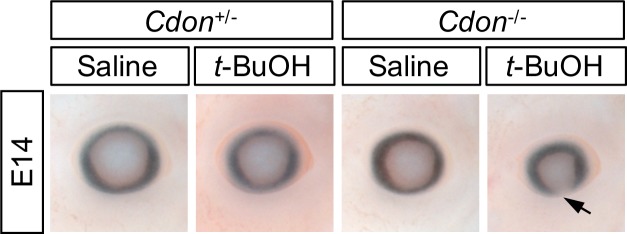
*t*-BuOH induces eye defects in *Cdon* mutant mice. *t*-BuOH-treated *Cdon*^*-/-*^ mice displayed micropthalamia and/or ventral coloboma (arrow), whereas *t*-BuOH-treated *Cdon*^*+/-*^ mice and saline-treated mice of either genotype did not.

We next collected and analyzed embryos at E14.0 to assess additional features of HPE, including midfacial and forebrain defects. Similar to what we have seen with EtOH, *t*-BuOH-treated *Cdon*^*-/-*^ embryos developed externally visible HPE phenotypes, including fused upper lip and single nostril, while wild type and *Cdon*^*+/-*^ littermates were not affected. More than 50% of *t*-BuOH-treated *Cdon*^*-/-*^ embryos showed at least one external feature of HPE, similar to but slightly lower than the 65–70% seen at this stage with EtOH treatment (Fig 2, Table 2; [25, 42]). Interestingly, ~23% of *t*-BuOH-treated *Cdon*^*-/-*^ embryos also had coloboma and/or microphthalamia in one or both eyes (Fig 3, Table 2); these phenotypes were less common in EtOH-treated *Cdon*^*-/-*^ embryos (~6% [25]).

**Table 2:** Frequency of HPE defects in *t*-BuOH-treated mice at E14.

Defect	Treatment	Genotype (# affected/total (%))	
		*Cdon*^*+/-*^	*Cdon*^*-/-*^
Fused upper lip	Saline	0/21	0/29
	*t*-BuOH	1/43 (2.3%)	20/39 (51%)*
Single nostril	Saline	0/21	0/29
	*t*-BuOH	1/43 (2.3%)	7/39 (18%)**
Coloboma/microphthalamia	Saline	0/21	0/29
	*t*-BuOH	0/43	9/39 (23%)***
Lobar HPE	Saline	0/3	0/3
	*t*-BuOH	0/3^1^	2/4^2^
Defective palatogenesis	Saline	0/3	0/3
	*t*-BuOH	0/3^1^	4/4^2^
Diminished nasal septum	Saline	0/3	0/3
	*t*-BuOH	0/3^1^	4/4^2^

*p<0.0001

**p<0.05

***p<0.001 when comparing *t*-BuOH-treated *Cdon*^*-/-*^ embryos with saline-treated *Cdon*^*-/-*^ embryos with two-tailed Fisher’s exact test.

^1^These embryos did not have external HPE features.

^2^These embryos displayed external HPE features.

Four E14.0 *t*-BuOH-treated *Cdon*^*-/-*^ embryos with external HPE phenotypes and three embryos from each control group were sectioned and stained with Hematoxylin and Eosin (H&E). Lobar HPE, characterized by a partitioned forebrain that lacked ventral midline structure, was found in two out of four *t*-BuOH-treated *Cdon*^*-/-*^ embryos (Fig 2, Table 2). All four *t*-BuOH-treated *Cdon*^*-/-*^ embryos showed a narrower midface, lack of or diminished nasal septum, and defects in palate formation, including clefting (Fig 2, Table 2). These phenotypes and the frequency at which they were induced were very similar between EtOH- and *t*-BuOH-treated *Cdon*^*-/-*^ embryos [25, 42]. *t*-BuOH-treated *Cdon*^*+/-*^ embryos and saline-treated embryos of all genotypes showed normal developmental patterning of midline structures upon H&E staining (Fig 2, Table 2).

### Reduced expression of *Shh* and *Nkx2*.*1* in the forebrains of *t*-BuOH-treated *Cdon*^*-/-*^ embryos

**Fig 4:**
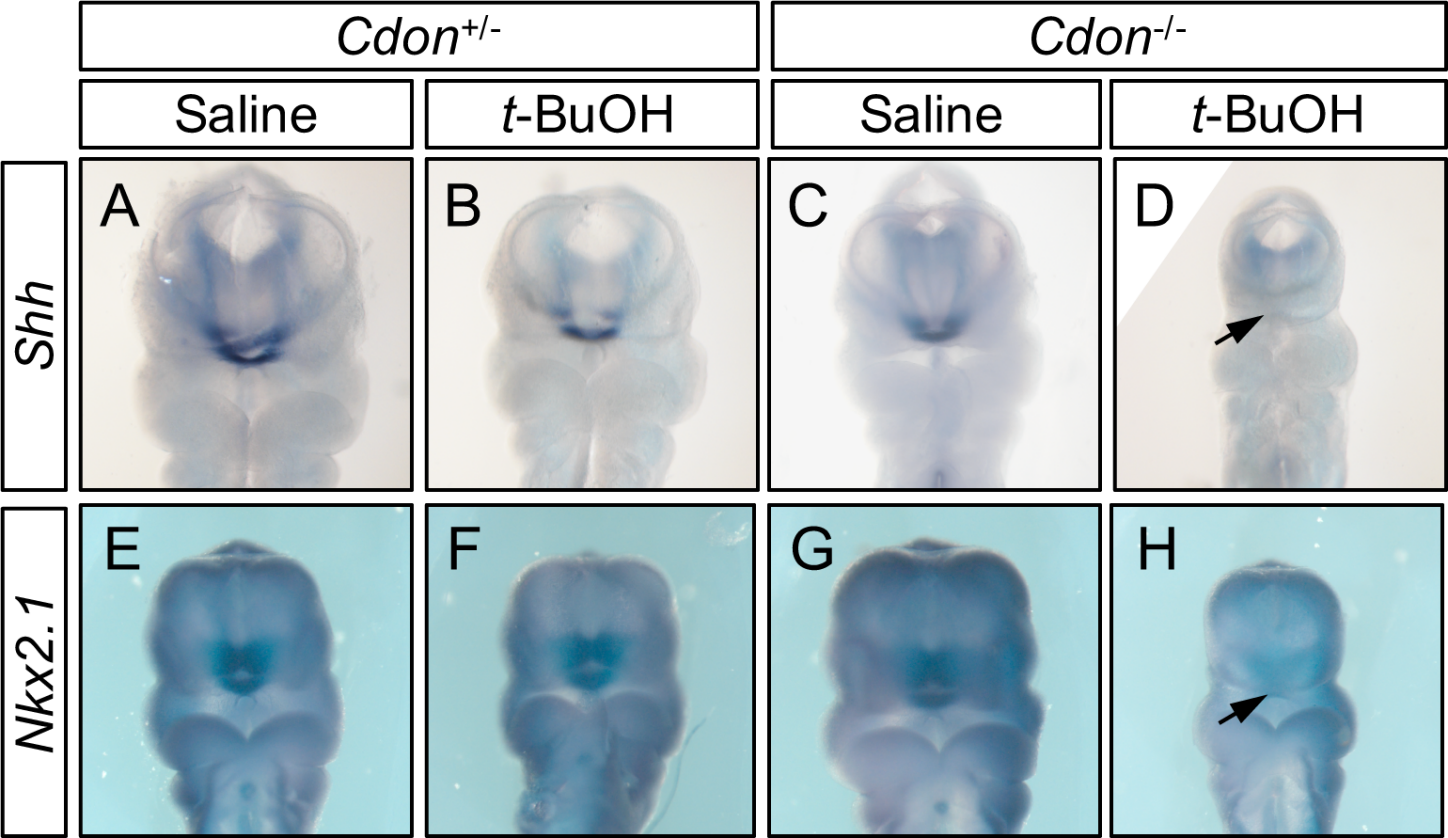
Reduced expression of *Shh* and *Nkx2*.*1* in forebrains of *t*-BuOH-treated *Cdon*^*-/-*^ mice. Whole mount in situ hybridization analysis of *Shh* (A-D) and *Nkx2*.*1* (E-H) expression in E10.25 embryos of the indicated genotype and treatment. Expression of *Shh* and *Nkx2*.*1* were specifically reduced in the rostroventral forebrain of *t*-BuOH-treated *Cdon*^*-/-*^ embryos (D and H, arrows). Three of four t-BuOH-treated *Cdon*^*-/-*^ embryos had diminished *Shh* expression and four of five embryos had reduced *Nxk2*.*1* expression.

Development of midline structures of the forebrain and midface is regulated by Shh pathway activity [43–48]. This occurs by a progressive mechanism, with a reiterative requirement for Shh signaling throughout rostroventral midline development, from early forebrain partitioning to fine patterning of the midface and palate; successful patterning of early midline structures is required for induction of *Shh* expression and pathway activation in midline structures that develop subsequently. The specificity of the Cdon mutation plus EtOH model, and the importance of Shh pathway signaling strength in EtOH-induced HPE, was demonstrated by the fact that removal of one copy of the negative regulator *Ptch1* rescued HPE in >75% of treated *Cdon*^*-/-*^ embryos [42]. Consistent with this notion, EtOH-treated *Cdon*^*-/-*^ embryos display reduced expression of *Shh* and Shh pathway target genes at various stages of rostroventral midline development [25, 42]. To examine whether *t*-BuOH treatment also did so, we assessed expression of *Shh* and the direct Shh pathway target gene, *Nkx2*.*1*, in the developing forebrain by whole mount in situ hybridization of E10.25 embryos. Expression of both genes was decreased specifically in the ventral forebrains of *t*-BuOH-treated *Cdon*^*-/-*^ embryos, but not any of the control embryos (Fig 4). As mentioned in the subsection above, we note that the *t*-BuOH-treated *Cdon*^*-/-*^ embryo shown in panel 3H is an example of one with a very severe HPE phenotype.

### Failure of antioxidants to rescue EtOH- or *t*-BuOH-induced HPE in *Cdon*^-/-^ mice

**Fig 5:**
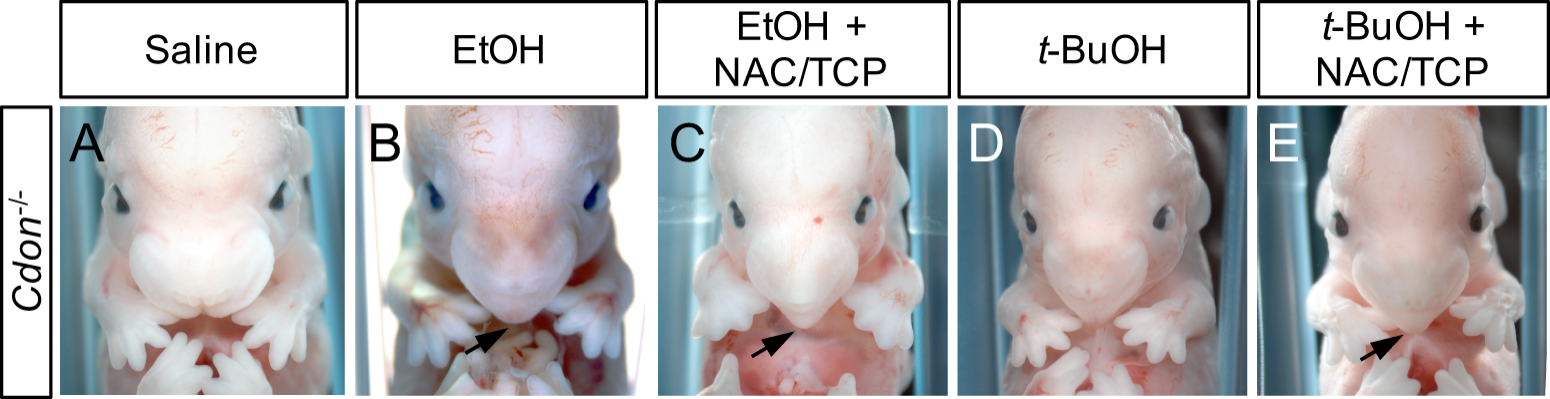
Failure of antioxidant treatment to rescue EtOH- or *t*-BuOH-induced HPE in *Cdon*^*-/-*^ mice. (A-E) Frontal views of E14 embryos. EtOH- and *t*-BuOH-treated *Cdon*^*-/-*^ embryos (B and D, respectively) developed strong facial features of HPE, including single nostril and smooth, pointed philtrum (arrows). These phenotypes were not rescued by treatment with N-acetylcysteine plus α-tocopherol (NAC/TCP) (C, E).

Administration of exogenous antioxidant compounds can often ameliorate the effects of pro-oxidant toxins. We therefore tested whether this might be true for EtOH-induced HPE in *Cdon*^*-/-*^ embryos. We selected an antioxidant regimen that had previously been demonstrated to be effective in reversing the effects in early pregnancy of the oxidant chemical, paraquat, in genetically-sensitized mice [40]. Pregnant females from intercrosses of *Cdon*^*+/-*^ mice were administered IP a combination of N-acetylcysteine (50 mg/kg) and α-tocopherol (1 g/kg) on days E5.0, E6.0, and E7.0, plus or minus the standard E7.0 treatment with EtOH. Embryos were collected at E14.0 and scored for external signs of HPE, including fused upper lip, single nostril, and coloboma/microphthalmia. The frequencies of these EtOH-induced phenotypes were unchanged by antioxidant treatment (Fig 5, Table 3). Similarly, N-acetylcysteine/α-tocopherol administration did not prevent *t*-BuOH-induced HPE (Fig 5, Table 4).

**Table 3:** Frequency of HPE defects in EtOH- plus antioxidant-treated mice at E14.

Defect	*Cdon*^*+/-*^			*Cdon*^*-/-*^		
	Saline	EtOH	EtOH +NAC/TCP*	Saline	EtOH	EtOH +NAC/TCP**
Fused upper lip	0/48	0/33	0/10	1/22	13/18	15/20
Single nostril	0/48	0/33	0/10	1/22	5/18	5/20
Coloboma/ microphthalamia	0/48	0/33	0/10	0/22	1/18	4/20

*NAC, N-acetylcysteine; TCP, α-tocopherol

**Frequencies of EtOH-induced HPE defects were not significantly altered by NAC/TCP.

**Table 4:** Frequency of HPE defects in *t*-BuOH- plus antioxidant-treated mice at E14.

Defect	*Cdon*^*+/-*^		*Cdon*^*-/-*^	
	*t*-BuOH	*t*-BuOH +NAC/TCP*	*t*-BuOH	*t*-BuOH +NAC/TCP**
Fused upper lip	1/43	0/12	20/39	8/15
Single nostril	1/43	0/21	7/39	3/15
Coloboma/ microphthalamia	0/43	0/21	9/39	3/15

*NAC, N-acetylcysteine; TCP, α-tocopherol

**Frequencies of *t*-BuOH-induced HPE defects were not significantly altered by NAC/TCP.

**Fig 6:**
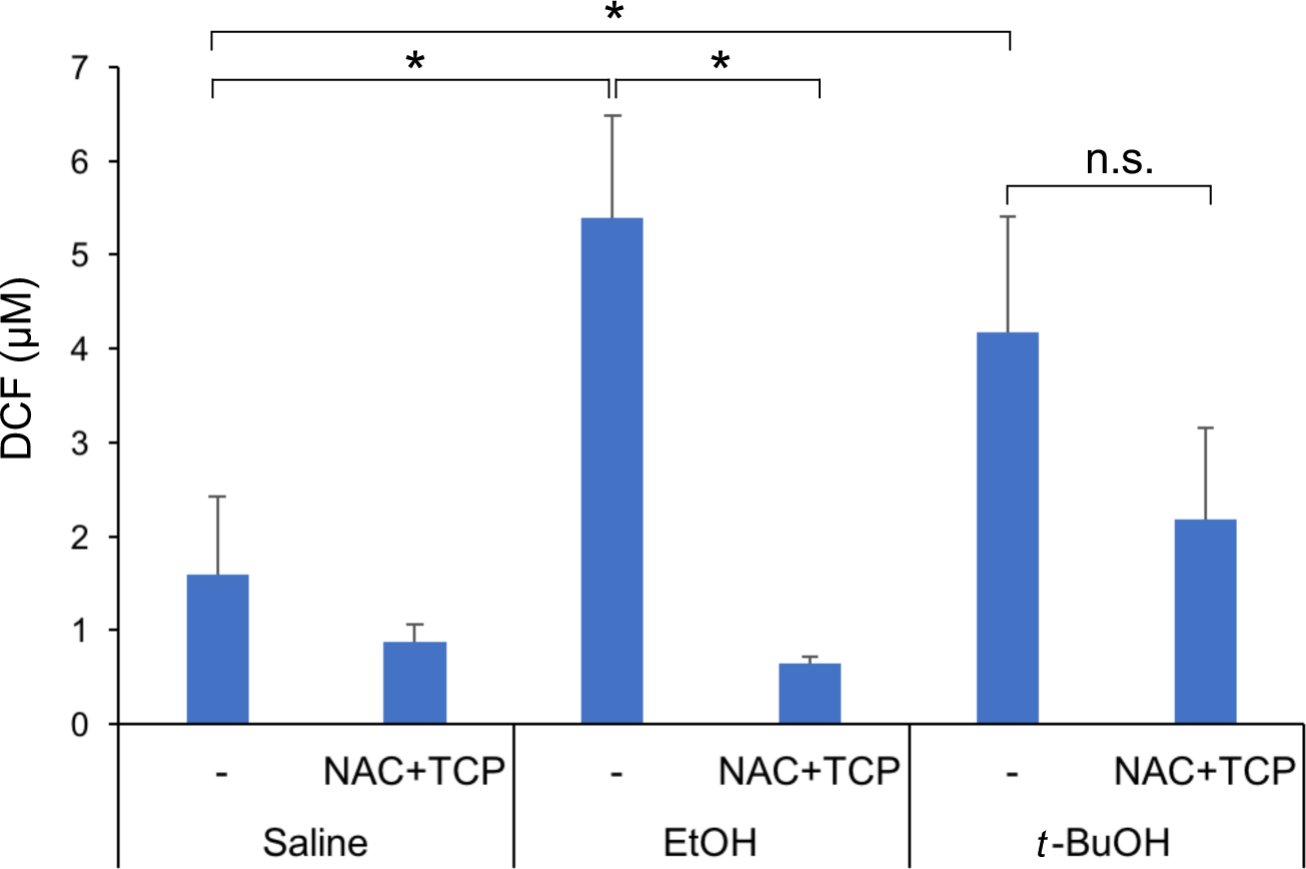
ROS/RNS levels in livers of mice from various treatment groups. ROS/RNS levels were measured by production of the fluorescent compound, DCF. EtOH and *t*-BuOH both increased ROS/RNS levels, relative to the saline control. N-acetylcysteine plus α-tocopherol (NAC+TCP) treatment normalized ROS/RNS levels in livers after EtOH exposure, but not after *t*-BuOH exposure. *p<0.05 with two-tailed Fisher’s exact test; n.s., not significant; values are means ± SD, n = 3–4 mice per point.

**Fig 7:**
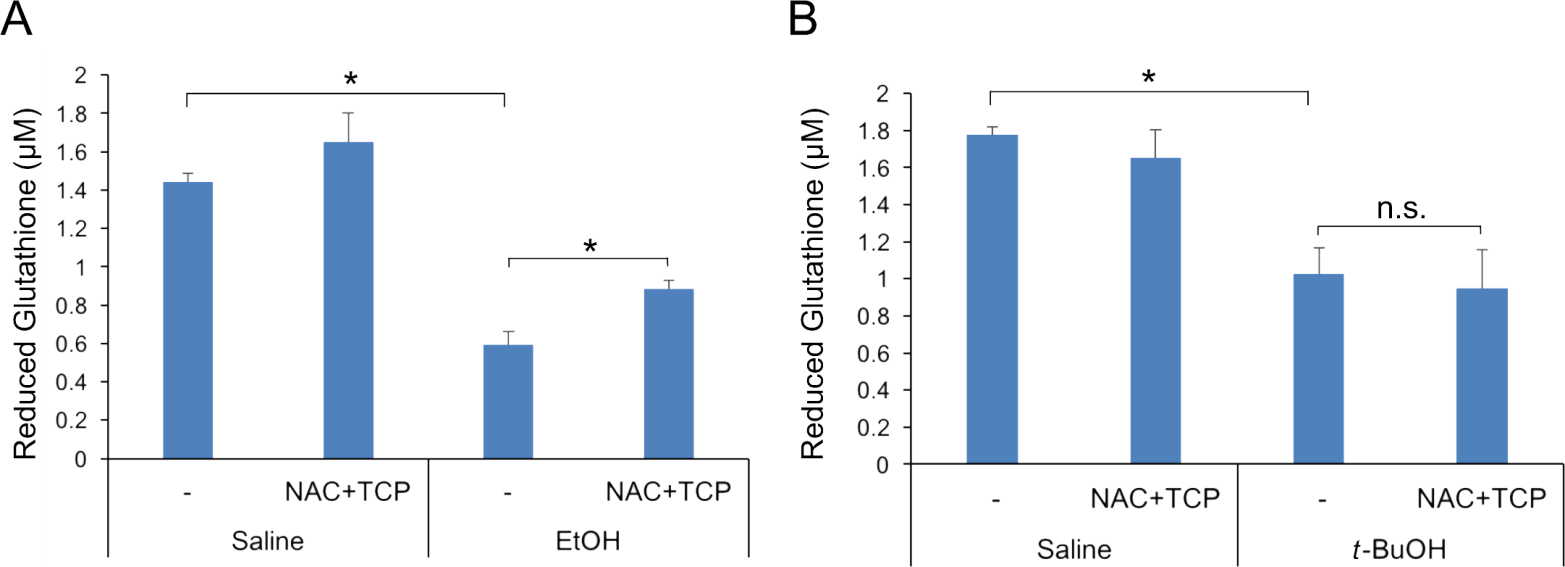
Reduced glutathione levels in livers of mice from various treatment groups. (A, B) Reduced glutathione (GSH) levels were analyzed 12 hours after EtOH (A) or *t*-BuOH (B) exposure. EtOH and *t*-BuOH both decreased GSH levels, relative to the saline control. N-acetylcysteine plus α-tocopherol (NAC+TCP) treatment partially rescued GSH levels in livers after EtOH exposure (A), but not after *t*-BuOH exposure (B). *p<0.05 with two-tailed Fisher’s exact test; n.s., not significant; values are means ± SD, n = 3–4 mice per point.

To confirm that the antioxidant treatment relieved oxidant stress induced by EtOH, we employed a DCF fluorescence assay to measure levels of reactive oxygen and nitrogen species (ROS/RNS) in the livers of female mice 12 hours after treatment with EtOH. EtOH increased the levels of ROS/RNS by ~3.3-fold over that seen in saline-treated control mice (Fig 6). Co-administration of N-acetylcysteine/α-tocopherol with EtOH reduced these levels back to that seen in the controls. *t*-BuOH-treated mice also had significantly elevated levels of ROS/RNS (Fig 6). However, N-acetylcysteine/α-tocopherol administration was much less effective at normalizing ROS/RNS levels in *t*-BuOH-treated mice than in EtOH-treated animals. Although ROS/RNS levels trended lower in *t*-BuOH-treated mice co-administered the antioxidants, this was not statistically significant (Fig 6). We also measured levels of reduced glutathione (GSH) in livers of treated female mice. EtOH decreased GSH levels by ~60%, as compared to saline-treated mice (Fig 7). N-acetylcysteine/α-tocopherol administration increased GSH levels by ~50% in EtOH-treated mice, relative to EtOH treatment alone, whereas it did not have a significant effect on GSH levels in the saline-treated control mice (Fig 7). Again, results with GSH measurements in *t*-BuOH-treated mice differed from those with EtOH-treated mice (Fig 7). *t*-BuOH decreased GSH levels by ~40%, less than that seen with EtOH. In contrast to the findings with EtOH, N-acetylcysteine/α-tocopherol treatment had no effect on the *t*-BuOH-induced reduction in GSH levels. Therefore, N-acetylcysteine/α-tocopherol treatment re-normalized ROS/RNS levels and restored GSH levels to a significant extent, without altering the frequency of EtOH-induced HPE.

## Discussion

EtOH is a human teratogen [15]. The range of effects in individuals exposed in utero to EtOH is broad and referred to under the umbrella term, fetal alcohol spectrum disorders (FASD). FASD can include a variety of structural and behavioral deficits [49]. Among the developmental defects that EtOH has been implicated in is HPE, the most common defect of forebrain and midface development [1]. Several mechanisms have been proposed to underlie EtOH’s varied teratogenic roles. Among these, some require its oxidative metabolism, while others do not. For example, metabolism by ADH and Cyp2E1 produces acetaldehyde, which is important for EtOH-induced exencephaly [28]. Additionally, it has been proposed that EtOH and acetaldehyde could act as competitive inhibitors during the enzymatic synthesis of RA from retinol (which involves ADH and ALDH reactions), resulting in developmental defects due to RA deficiency [29–31]. Moreover, EtOH metabolism leads to production of a variety of oxidant species, producing an overall condition of oxidative stress [26, 27]. On the other hand, EtOH itself has solvent properties and can perturb biological membranes [32–34]. EtOH also binds directly to the Ig cell adhesion molecule, L1, and interferes with its adhesive function [35, 36]. It seems likely that the multiple teratogenic outcomes associated with fetal alcohol exposure may be caused by distinct mechanisms, depending on the specific developmental defect in question.

To address how EtOH induces HPE, we used a mouse model that displays high specificity and fidelity to human HPE. *Cdon* mutation and in utero EtOH exposure synergize to produce a full range of HPE spectrum defects, with high penetrance [25]. We used this model to address the roles of EtOH metabolism and associated oxidative stress in HPE. We found that *t*-BuOH, a branched chain alcohol that is neither a substrate for, nor inhibitor of, ADH or Cyp2E1, was a potent inducer of HPE in *Cdon*^*-/-*^ mice. Furthermore, *t*-BuOH induced a quantitatively and qualitatively similar response to EtOH at less than one-fifth the dose required for EtOH. These findings are consistent with the idea that it is EtOH itself, rather than its metabolism, which is required to induce HPE. One reason for *t*-BuOH’s greater potency is likely that, in the case of EtOH, the teratogen itself is depleted by ADH-dependent metabolism, whereas this does not occur with *t*-BuOH. *t*-BuOH is, however, metabolized by phase II enzymes and excreted (mainly as a sulfate conjugate), so it may also simply be a more efficient teratogen than EtOH. In addition to indicating that oxidative metabolism is probably unnecessary for EtOH-induced HPE, our findings also suggest that competitive inhibition of retinol metabolism is not likely to be a major mechanism of EtOH-induced HPE either. It is worth noting in this regard that mice lacking *RDH10* or *Raldh2*, important enzymes in RA synthesis, have severe developmental defects but do not appear to have overt HPE [50–52]. Additionally, it has been concluded that the forebrain and facial defects seen in chick embryos treated with retinoid receptor antagonists are distinct from HPE [53].

In a second approach, we found that administration of the antioxidants N-acetylcysteine/α-tocopherol prior to and during EtOH or *t*-BuOH treatment did not alter the frequency or severity of HPE phenotypes. EtOH induced oxidative stress in mice, as evidenced by an increase in ROS/RNS levels and a reduction of GSH levels in the livers of treated female mice. N-acetylcysteine/α-tocopherol treatment renormalized ROS/RNS levels, despite its lack of effect on EtOH-induced HPE. It also restored GSH levels, though not fully to the levels seen in untreated mice. These results argue that EtOH-induced oxidative stress can be segregated from its action as an HPE-inducing teratogen. It is difficult to accurately assess oxidative stress directly in the very early embryos that are exposed to EtOH, so we cannot fully rule out that the failure of antioxidant treatment to alter EtOH-induced HPE was due to an insufficient reversal of oxidant stress in the embryos themselves. Nevertheless, the EtOH-induced increase in liver ROS/RNS levels was returned to baseline with N-acetylcysteine/α-tocopherol treatment. Furthermore, the antioxidant regimen used in these experiments was previously found to attenuate the effects of paraquat on embryo implantation in genetically sensitized mice [40]. Unlike EtOH, paraquat is thought to work exclusively via redox cycling and oxidative stress [54].

*t*-BuOH treatment also increased ROS/RNS levels and depressed GSH levels. However, administration of N-acetylcysteine/α-tocopherol did not rescue ROS/RNS or GSH levels in *t*-BuOH-treated mice. Given that EtOH is oxidatively metabolized and *t*-BuOH is not, the mechanisms of EtOH- vs. *t*-BuOH-induced ROS/RNS production, GSH depletion, and, presumably, transient liver toxicity, may be different. It must be emphasized that despite these differences between EtOH and *t*-BuOH, each alcohol induced a very similar penetrance and spectrum of HPE phenotypes at the doses studied, arguing that the mechanism(s) that underlie their HPE teratogencity are similar. Taking all the results together, we conclude that EtOH’s teratogenic effects in HPE are unlikely to involve oxidative metabolism and that oxidative stress itself may not be a critical component of its action in inducing HPE.

In utero EtOH exposure in C57BL/6J mice induces HPE with low penetrance [55, 56]. In contrast to our findings, Aoto et al. reported that feeding these mice a diet supplemented with α-tocopherol prevented EtOH-induced HPE [55]. However, this system used very high levels of α-tocopherol (5% of the diet). While α-tocopherol has antioxidant properties, it may also function as a membrane stabilizer [57–59]. Given our findings in this study, we suggest that α-tocopherol may have acted in this latter manner, at least in part, to prevent EtOH-induced HPE in C57BL/6J mice. It should be noted that prenatal antioxidant treatment is under consideration as a means of reversing or preventing EtOH’s teratogenicity in FASD [60]. Our results argue that this may be ineffective in HPE, and perhaps some other aspects of FASD.

If EtOH and *t*-BuOH are actual, rather than proximal, teratogens in HPE, what might be their mechanism of teratogenicity? EtOH is known to perturb membranes through solvent-like effects, and it seems likely that *t*-BuOH would be even more effective at this. Such a mechanism would also be consistent with the observation that high levels of α-tocopherol prevented HPE when the combination of N-acetylcysteine/α-tocopherol did not (albeit in different mouse strains). Membrane perturbation might in turn disrupt assembly or stability of membrane-associated signaling complexes that regulate rostroventral midline development during gastrulation, the time of sensitivity to EtOH-induced HPE. It is worth noting that the window of sensitivity to EtOH-induced HPE is very narrow, starting at about E7.0 and over by E7.5 ([25, 55] and our unpublished results). This is a time of rapid and extreme morphogenetic change that could be particularly susceptible to such effects. Finally, it is also possible that EtOH and *t*-BuOH could exert their effects via interaction with specific proteins, analogous to EtOH’s effects on the L1 cell adhesion molecule. It seems unlikely that L1 itself is the target in HPE, however, as mutations in L1 in humans and mice are not known to be associated with HPE [61].

In summary, we report findings consistent with the notion that EtOH itself, rather than a consequence of its oxidative metabolism, acts as an HPE-inducing teratogen. We emphasize that other developmental defects associated with in utero EtOH exposure at different stages of development may arise from different mechanisms that do involve EtOH metabolism and oxidative stress [62, 63]. Use of a highly specific model of HPE allowed a specific focus on this defect, and it will be informative to apply these approaches to other systems with distinct EtOH-related outcomes.
